# Supplementary material for: Splice-Junction-Based Mapping of Alternative Isoforms in the Human Proteome
Source: Cell Rep. Author manuscript; Available in PMC 2020 Jan 15. (PMC6961840; doi:10.1016/j.celrep.2019.11.026)
Supplement: 2 [file NIHMS1546469-supplement-2.zip › DF1/PXD010154/Heart/Heart_85_VPS52_KHKPNTPGFLSSAAR.pdf]

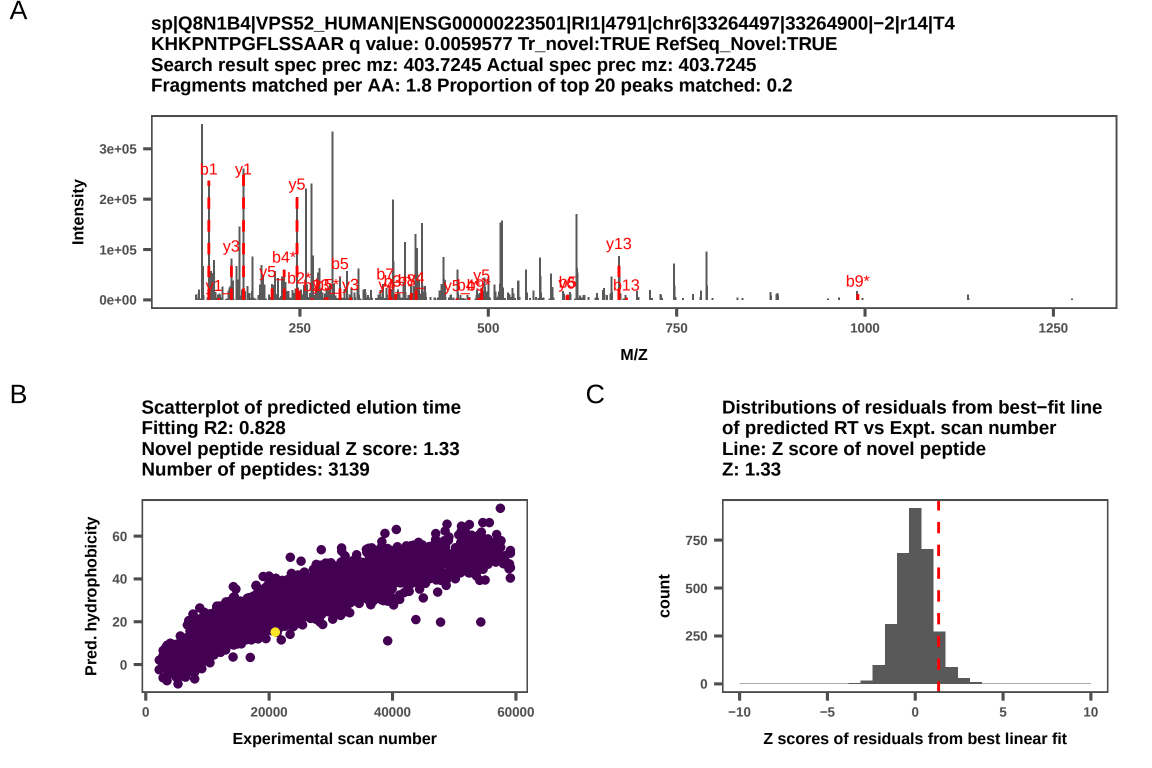
A
sp|Q8N1B4|VPS52_HUMAN|ENSG00000223501|RI1|4791|chr6|33264497|33264900|−2|r14|T4
KHKPNTPGFLSSAAR q value: 0.0059577 Tr_novel:TRUE RefSeq_Novel:TRUE
Search result spec prec mz: 403.7245 Actual spec prec mz: 403.7245
Fragments matched per AA: 1.8 Proportion of top 20 peaks matched: 0.2
Intensity
3e+05
2e+05
1e+05
0e+00
b1
y1
y5
y3
b4*
b5
y5
b2*
y13
b13
b9*
250
500
750
1000
1250
M/Z
B
Scatterplot of predicted elution time
Fitting R2: 0.828
Novel peptide residual Z score: 1.33
Number of peptides: 3139
Pred. hydrophobicity
60
40
20
0
0
20000
40000
60000
Experimental scan number
C
Distributions of residuals from best−fit line
of predicted RT vs Expt. scan number
Line: Z score of novel peptide
Z: 1.33
count
750
500
250
0
−10
−5
0
5
10
Z scores of residuals from best linear fit
